# Supplementary material for: Validation and Demonstration of an Atmosphere-Temperature-pH-Controlled Stirred Batch Reactor System for Determination of (Nano)Material Solubility and Dissolution Kinetics in Physiological Simulant Lung Fluids
Source: Nanomaterials (Basel). 2022 Feb 2;12(3):517. doi: 10.3390/nano12030517 (PMC8838572; doi:10.3390/nano12030517)
Supplement: Supplementary file 1 [file nanomaterials-12-00517-s001.zip › nanomaterials-1530714-supplementary.pdf]

Supplementary Materials

Validation and Demonstration of an Atmosphere-Temperature-pH-Controlled Stirred Batch Reactor System for Determination of (Nano)Material Solubility and Dissolution Kinetics in Physiological Simulant Lung Fluids

Else Holmfred [1,2,*], Katrin Loeschner [2], Jens J. Sloth [2] and Keld Alstrup Jensen [1,*]

[1] National Research Centre for the Working Environment, 2100 Copenhagen, Denmark
[2] Research Group for Analytical Food Chemistry, Division of Food Technology, National Food Institute, Technical University of Denmark, 2800 Kgs. Lyngby, Denmark; kals@food.dtu.dk (K.L.); jjsl@food.dtu.dk (J.J.S.)
* Correspondence: elshol@food.dtu.dk (E.H.); kaj@nfa.dk (K.A.J.)

Table S1. ICP-MS parameters used during the analysis of dissolved ions and limit of detection in phagolysosomal fluid simulant.

Parameter [unit]	Al_2O_3	TiO_2 (NM-104)	ZnO (NM-110, 111, 113)	SiO_2 (NM-200)	CeO_2 (NM-212)	Bentonite (NM-600)
Nebulizer gas flow rate [mL/min]	1.01	1.05	1.03	1.04	1.03	1.05
Helium cell gas flow rate [mL/min]	4.55	4.35	4.35	4.43	n/a	4.29
Monitored isotopes [m/z]	^{27}Al	^{27}Al, ^{48}Ti	^{66}Zn	^{28}Si	^{140}Ce	^{27}Al, ^{28}Si
Internal standard [m/z]	^{103}Rh	^{103}Rh	^{103}Rh	^{89}Y	^{103}Rh	^{103}Rh
Limit of detection of the measured ion(s)	12 µg/L	Al: 5.2 µg/L Ti: 8.6 µg/L	70 µg/L	271 µg/L	0.03 µg/L	Al: 4.1 µg/L Si: 17 µg/L
Dilution factor for ICP-MS analysis	x10	x10	x1000	x100	x4	x10

Table S2. ICP-MS parameters used during the analysis of dissolved ions and limit of detection in low-calcium Gamble's solution.

Parameter [unit]	Al_2O_3	TiO_2 (NM-104)	ZnO (NM-110, 111, 113)	SiO_2 (NM-200)	CeO_2 (NM-212)	Bentonite (NM-600)
Nebulizer gas flow rate [mL/min]	1.01	1.02	1.00	1.02	1.02	1.02
Helium cell gas flow rate [mL/min]	4.50	n/a, 4.50	n/a	4.50	n/a	4.59
Monitored isotopes [m/z]	^{27}Al	^{27}Al, ^{49}Ti	^{66}Zn	^{28}Si	^{140}Ce	^{27}Al, ^{28}Si
Internal standard [m/z]	^{103}Rh	^{89}Y	^{103}Rh	^{75}As	^{103}Rh	^{103}Rh
Limit of detection of the measured ion(s)	28 µg/L	Al: 32 µg/L Ti: 1.1 µg/L	3.4 µg/L	4.7 µg/L	0.8 µg/L	Al: 5.7 µg/L Si: 168 µg/L,
Dilution factor for ICP-MS analysis	x10	x4	x200	x100	x2	x10

It should be noted that the LOD for Zn in low-calcium Gamble's solution is high compared to the determined LOD of Zn in PSF. This is mainly due to Zn impurities in chemicals used for preparation of the low-calcium Gamble´s solution. The concentration of dissolved Zn during 24 hours dissolution is approx. 200 times higher compared to LOD and is therefore not influenced by the relatively high LOD of Zn in low-calcium Gamble's solution.

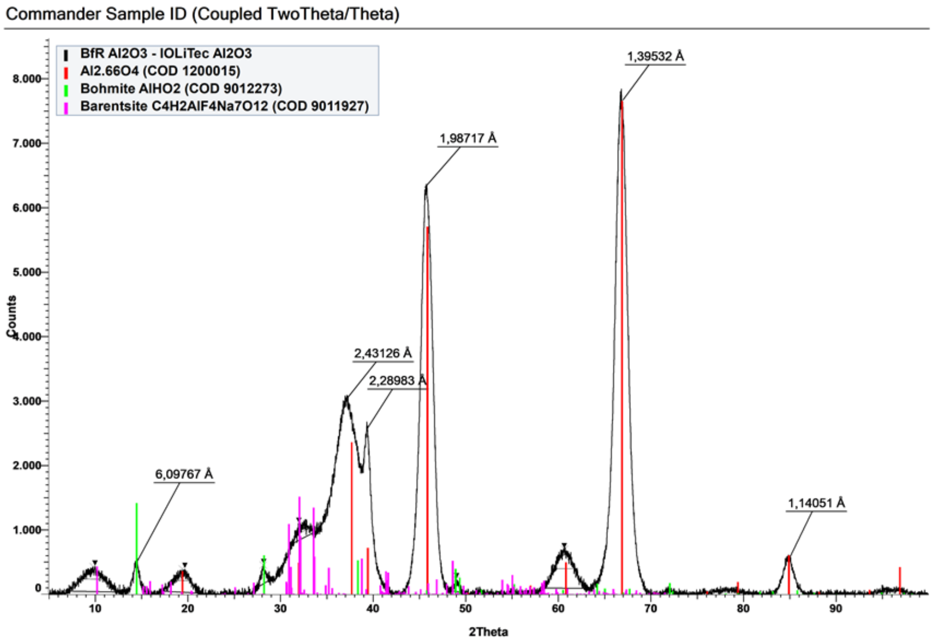

Figure S1. Powder X-ray diffraction spectrum of ɣ-Al_2O_3 showing presence of minor boehmite and potentially also barentsite.

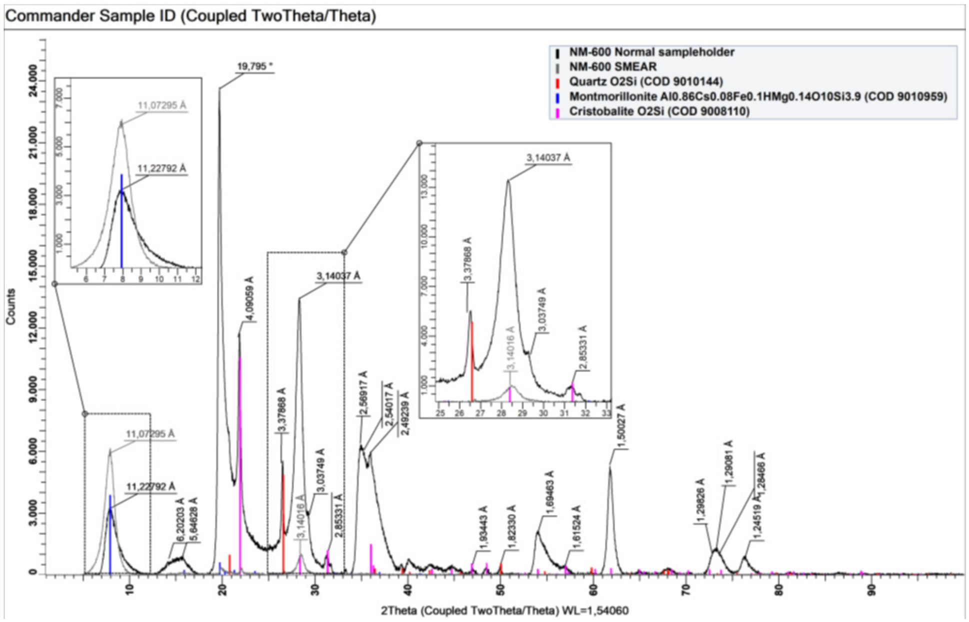

Figure S2. Powder X-ray diffraction spectra of bentonite (NM-600). The black-color spectrum is for bentonite (NM-600) while the grey-colored spectrum is obtained on a SMEAR. Montmorillonite, quartz and cristobalite were identified in the sample.

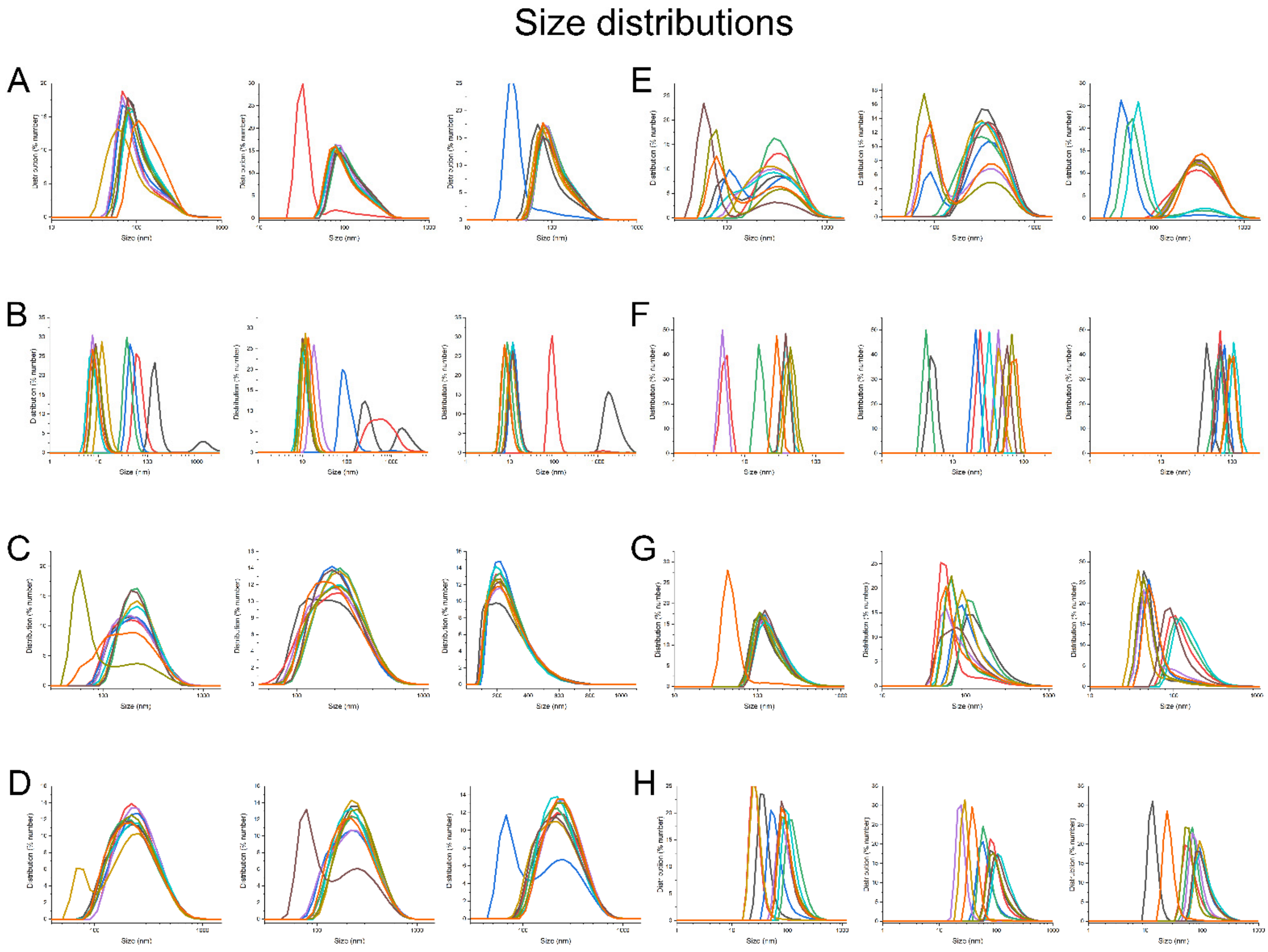

Figure S3. Size distributions (%number) of dispersions used for dissolution testing in low-calcium Gamble´s solution (n=3). The size distributions show the ten repeated measurements. A) Al_2O_3, B) TiO_2 (NM-104), C) ZnO (NM-110), D) ZnO (NM-111), E) ZnO (NM-113), F) SiO_2 (NM-200), G) CeO_2 (NM-212), and H) bentonite (NM-600).

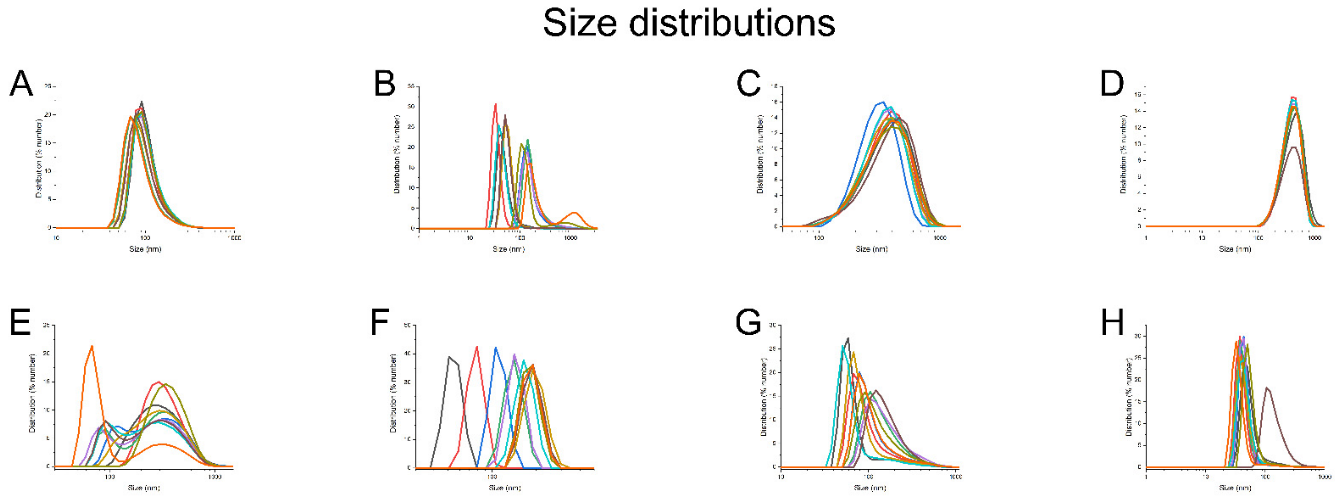

Figure S4. Size distributions (%number) of dispersions used for dissolution testing in phagolysosomal simulant fluid (n=3). The size distributions show the ten repeated measurements. A) Al_2O_3, B) TiO_2 (NM-104), C) ZnO (NM-110), D) ZnO (NM-111), E) ZnO (NM-113), F) SiO_2 (NM-200), G) CeO_2 (NM-212), and H) bentonite (NM-600).

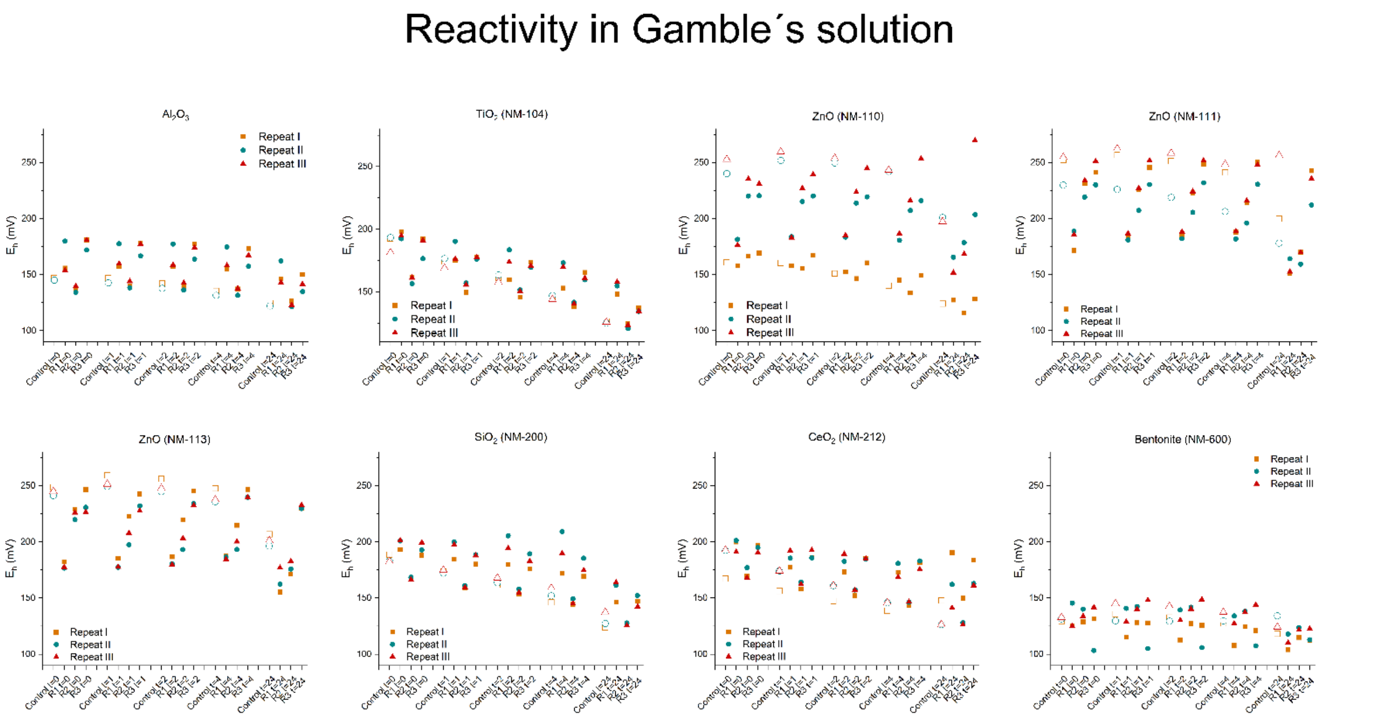

Figure S5. Reactivity of the test materials after testing in low-calcium Gamble´s solution. Upper row: Al_2O_3, TiO_2 (NM-104), ZnO (NM-110), and ZnO (NM-111). Lower row: ZnO (NM-113), SiO_2 (NM-200), CeO_2 (NM-212), and bentonite (NM-600). The control reactors (I, (□), II (○), and III (Δ)) represent the redox potential in the pure medium over 24 hours. Test reactors (I (■), II (●), and III (▲)) indicated as repeat I, repeat II, and repeat III represent the three reactors during each dissolution test.

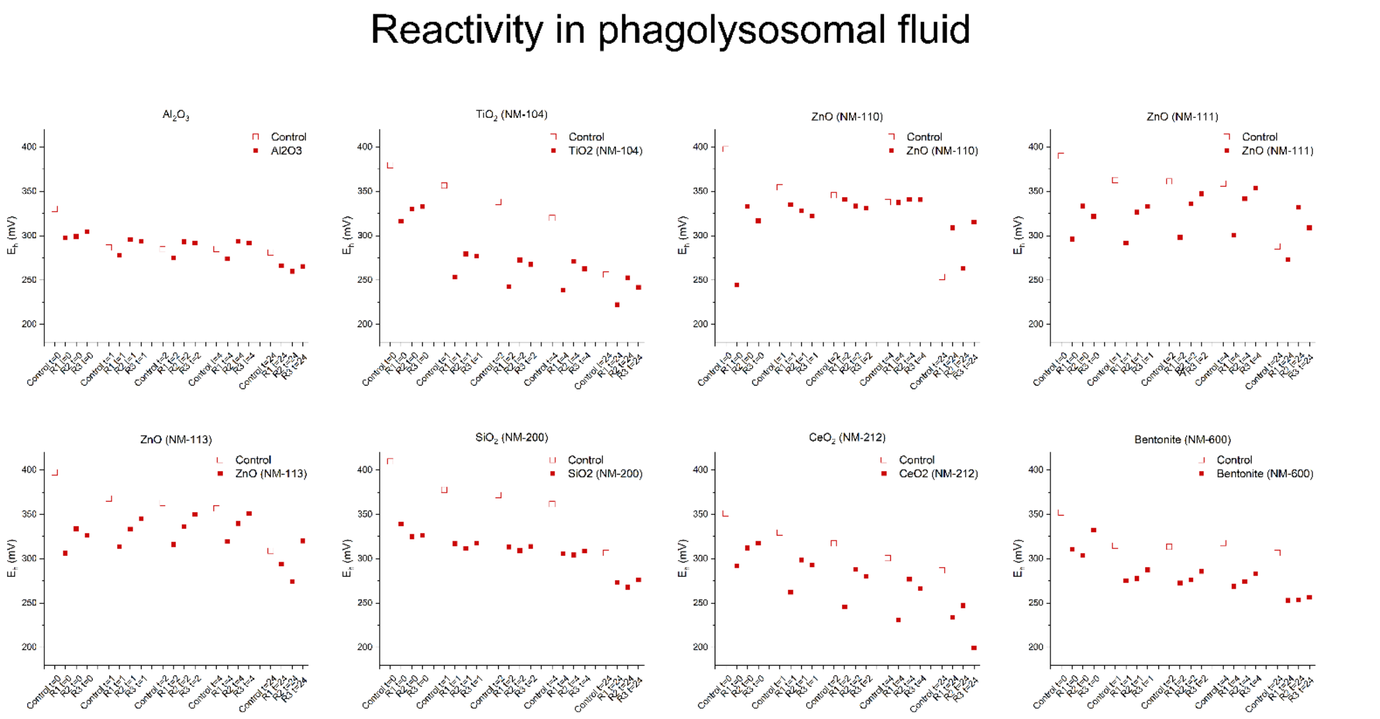

Figure S6. Reactivity of the test materials after testing in phagolysosomal fluid simulant. Upper row: Al_2O_3, TiO_2 (NM-104), ZnO (NM-110), and ZnO (NM-111). Lower row: ZnO (NM-113), SiO_2 (NM-200), CeO_2 (NM-212), and bentonite (NM-600). The control reactor (□) represents the redox potential in the pure medium over 24 hours. Test reactors (■) indicated as R1, R2, and R3 represents the three reactors during each dissolution test. .

Table S3. Measured redox potentials of Al_2O_3, TiO_2 (NM-104), ZnO (NM-110, NM-111, NM-113), SiO_2 (NM-200), CeO_2 (NM-212), and bentonite (NM-600) tested in low-calcium Gamble´s solution in triplicate (I, II, III).

**Repeat I: Al_2O_3 tested in Gamble´s solution**						
**Time [h]**	**Replicate [mV]**	**Replicate 1 [mV]**	**Replicate 2 [mV]**	**Replicate 3 [mV]**	**Average [mV]**	**Std.dev [mV]**
0	146.9	155.9	135.9	181.0	157.6	22.6
1	147.0	157.3	140.1	178.4	158.6	19.2
2	142.1	157.2	139.3	177.3	157.9	19.0
4	135.4	155.3	137.2	173.3	155.3	18.1
24	124.7	146.2	126.4	150.2	140.9	12.7
**Repeat II: Al_2O_3 tested in Gamble´s solution**						
**Time [h]**	**Replicate [mV]**	**Replicate 1 [mV]**	**Replicate 2 [mV]**	**Replicate 3 [mV]**	**Average [mV]**	**Std.dev [mV]**
0	145.1	179.9	134.3	172.0	162.1	24.3
1	142.7	177.7	138.3	166.7	160.9	20.3
2	137.8	177.3	136.3	163.9	159.2	20.9
4	131.5	174.8	131.6	157.4	154.6	21.7
24	122.1	162.4	121.5	134.9	139.6	20.9
**Repeat III: Al_2O_3 tested in Gamble´s solution**						
**Time [h]**	**Replicate [mV]**	**Replicate 1 [mV]**	**Replicate 2 [mV]**	**Replicate 3 [mV]**	**Average [mV]**	**Std.dev [mV]**
0	164.5	153.8	139.8	180.9	158.2	20.9
1	158.3	159.8	144.1	177.2	160.4	16.6
2	150.5	158.7	142.7	174.2	158.5	15.8
4	140.5	158.2	137.6	167.3	154.4	15.2
24	125.3	142.9	122.9	141.6	135.8	11.2
**Repeat I: TiO_2 (NM-104) tested in Gamble´s solution**						
**Time [h]**	**Replicate [mV]**	**Replicate 1 [mV]**	**Replicate 2 [mV]**	**Replicate 3 [mV]**	**Average [mV]**	**Std.dev [mV]**
0	192.6	197.8	161.6	192.4	183.9	19.5
1	174.2	175.0	149.6	177.7	167.4	15.5
2	161.1	159.8	145.7	173.6	159.7	14.0
4	145.9	152.9	138.0	165.5	152.1	13.8
24	126.4	148.1	124.8	137.3	136.7	11.7
**Repeat II: TiO_2 (NM-104) tested in Gamble´s solution**						
**Time [h]**	**Replicate [mV]**	**Replicate 1 [mV]**	**Replicate 2 [mV]**	**Replicate 3 [mV]**	**Average [mV]**	**Std.dev [mV]**
0	193.3	192.4	156.4	176.5	175.1	18.0
1	176.50	190.2	157.0	176.1	174.4	16.7
2	163.4	183.5	151.5	169.5	168.2	16.0
4	147.0	173.1	141.6	159.6	158.1	15.8
24	124.9	154.7	120.7	134.3	136.6	17.1
**Repeat III: TiO_2 (NM-104) tested in Gamble´s solution**						
**Time [h]**	**Replicate [mV]**	**Replicate 1 [mV]**	**Replicate 2 [mV]**	**Replicate 3 [mV]**	**Average [mV]**	**Std.dev [mV]**
0	181.3	194.7	161.2	191.0	182.3	18.4
1	168.9	176.2	155.6	177.5	169.8	12.3
2	157.6	173.8	150.3	170.8	165.0	12.8

4	143.9	169.8	140.4	160.9	157.0	15.1
24	125.8	157.9	123.4	134.6	138.6	17.6

Repeat I: ZnO (NM-110) tested in Gamble´s solution

Time [h]	Replicate [mV]	Replicate 1 [mV]	Replicate 2 [mV]	Replicate 3 [mV]	Average [mV]	Std.dev [mV]
0	160.9	157.9	166.7	169.2	164.6	5.94
1	160.4	157.8	155.6	167.2	160.2	6.16
2	151.0	152.5	146.6	160.6	153.2	7.03
4	140.2	145.1	134.0	149.5	142.9	7.99
24	124.1	127.4	115.9	128.3	123.9	6.91

Repeat II: ZnO (NM-110) tested in Gamble´s solution

Time [h]	Replicate [mV]	Replicate 1 [mV]	Replicate 2 [mV]	Replicate 3 [mV]	Average [mV]	Std.dev [mV]
0	240.1	181.4	220.2	220.6	207.4	22.5
1	251.9	183.9	215.3	220.2	206.5	19.7
2	249.7	183.4	213.9	219.4	205.6	19.4
4	242.1	180.6	207.4	216.1	201.4	18.5
24	201.0	165.6	178.6	203.6	182.6	19.3

Repeat III: ZnO (NM-110) tested in Gamble´s solution

Time [h]	Replicate [mV]	Replicate 1 [mV]	Replicate 2 [mV]	Replicate 3 [mV]	Average [mV]	Std.dev [mV]
0	252.9	176.4	235.4	231.1	214.3	32.9
1	259.6	183.0	227.1	239.5	216.5	29.7
2	253.9	185.1	223.7	245.0	217.9	30.4
4	243.3	186.7	216.1	253.5	218.8	33.5
24	197.3	151.8	168.4	269.8	196.7	63.9

Repeat I: ZnO (NM-111) tested in Gamble´s solution

Time [h]	Replicate [mV]	Replicate 1 [mV]	Replicate 2 [mV]	Replicate 3 [mV]	Average [mV]	Std.dev [mV]
0	252.3	171.6	231.6	241.2	214.8	37.7
1	256.7	183.1	225.8	245.7	218.2	32.0
2	251.4	185.5	223.0	248.7	219.1	31.8
4	241.3	187.9	214.3	250.3	217.5	31.3
24	199.7	150.8	170.0	242.7	187.8	48.5

Repeat II: ZnO (NM-111) tested in Gamble´s solution

Time [h]	Replicate [mV]	Replicate 1 [mV]	Replicate 2 [mV]	Replicate 3 [mV]	Average [mV]	Std.dev [mV]
0	229.9	188.7	219.2	230.2	212.7	21.5
1	226.0	181.0	207.3	230.4	206.2	24.7
2	218.9	182.1	205.6	232.0	206.6	25.0
4	206.4	181.8	196.1	230.6	202.8	25.1
24	178.1	164.2	159.3	212.3	178.6	29.3

Repeat III: ZnO (NM-111) tested in Gamble´s solution

Time [h]	Replicate [mV]	Replicate 1 [mV]	Replicate 2 [mV]	Replicate 3 [mV]	Average [mV]	Std.dev [mV]
0	254.7	185.5	234	251.3	223.6	34.1
1	262.4	186.8	227.3	251.8	222.0	32.8
2	258.4	188.3	224.2	251.8	221.4	31.8

4	248.3	188.8	216.0	248.1	217.6	29.7
24	256.3	152.6	169.8	235.7	186.0	43.9

Repeat I: ZnO (NM-113) tested in Gamble´s solution

Time [h]	Replicate [mV]	Replicate 1 [mV]	Replicate 2 [mV]	Replicate 3 [mV]	Average [mV]	Std.dev [mV]
0	248.8	182.2	228.9	246.5	219.2	33.2
1	259.2	185.3	223.0	242.6	217.0	29.1
2	256.2	186.7	219.9	245.2	217.3	29.3
4	247.6	187.9	214.7	246.7	216.4	29.4
24	206.8	155.5	171.4	230	185.6	39.2

Repeat II: ZnO (NM-113) tested in Gamble´s solution

Time [h]	Replicate [mV]	Replicate 1 [mV]	Replicate 2 [mV]	Replicate 3 [mV]	Average [mV]	Std.dev [mV]
0	241.2	176.7	219.8	230.8	209.1	28.6
1	249.3	177.3	197.4	232.1	202.3	27.7
2	244.8	180.4	193.2	234.0	202.5	28.0
4	235.8	186.5	193.3	239.8	206.5	29.0
24	196.3	162.4	175.9	229.7	189.3	35.6

Repeat III: ZnO (NM-113) tested in Gamble´s solution

Time [h]	Replicate [mV]	Replicate 1 [mV]	Replicate 2 [mV]	Replicate 3 [mV]	Average [mV]	Std.dev [mV]
0	244.9	177.5	226	226.5	210.0	28.1
1	251.5	177.7	207.9	227.7	204.4	25.2
2	247.6	179.7	203.2	232.5	205.1	26.5
4	237.5	184.6	200.3	239.7	208.2	28.4
24	201.1	177.0	182.8	232.8	197.5	30.7

Repeat I: SiO_2 (NM-200) tested in Gamble´s solution

Time [h]	Replicate [mV]	Replicate 1 [mV]	Replicate 2 [mV]	Replicate 3 [mV]	Average [mV]	Std.dev [mV]
0	188.7	193.4	168.0	188.2	183.2	13.4
1	173.5	184.5	158.8	180.2	174.5	13.8
2	161.7	180.1	153.5	175.9	169.8	14.3
4	146.2	171.9	144.5	169.3	161.9	15.1
24	124.1	146.6	126.4	147.2	140.1	11.8

Repeat II: SiO_2 (NM-200) tested in Gamble´s solution

Time [h]	Replicate [mV]	Replicate 1 [mV]	Replicate 2 [mV]	Replicate 3 [mV]	Average [mV]	Std.dev [mV]
0	183.1	200.9	168.5	192.9	187.4	16.9
1	172.1	199.9	161.1	188.5	183.2	19.9
2	163.7	205.5	157.8	189.4	184.2	24.3
4	152.0	209.1	149.3	185.5	181.3	30.1
24	127.4	161.5	127.8	152.3	147.2	17.4

Repeat III: SiO_2 (NM-200) tested in Gamble´s solution

Time [h]	Replicate [mV]	Replicate 1 [mV]	Replicate 2 [mV]	Replicate 3 [mV]	Average [mV]	Std.dev [mV]
0	182.4	201.4	166.3	199.4	189.0	19.7
1	174.8	197.5	159.5	187.9	181.6	19.8
2	167.9	194.4	154.8	182.9	177.4	20.4

4	158.9	189.8	145.0	174.7	169.8	22.8
24	137.3	163.9	125.6	142.4	144.0	19.2

Repeat I: CeO_2 (NM-212) tested in Gamble´s solution

Time [h]	Replicate [mV]	Replicate 1 [mV]	Replicate 2 [mV]	Replicate 3 [mV]	Average [mV]	Std.dev [mV]
0	167.3	200.0	169.4	196.8	188.7	16.8
1	156.2	177.4	158.2	186.1	173.9	14.3
2	147.4	173.4	152.2	185.6	170.4	16.9
4	138.8	173.1	143.3	181.5	166.0	20.1
24	147.9	190.4	150.0	184.0	174.8	21.7

Repeat II: CeO_2 (NM-212) tested in Gamble´s solution

Time [h]	Replicate [mV]	Replicate 1 [mV]	Replicate 2 [mV]	Replicate 3 [mV]	Average [mV]	Std.dev [mV]
0	192.6	201.2	177.1	194.9	191.1	12.5
1	174.2	185.5	164.1	185.9	178.5	12.5
2	161.1	182.7	157.2	184.6	174.8	15.3
4	145.9	180.9	146.3	183.1	170.1	20.6
24	126.4	162.3	128.0	162.9	151.1	20.0

Repeat III: CeO_2 (NM-212) tested in Gamble´s solution

Time [h]	Replicate [mV]	Replicate 1 [mV]	Replicate 2 [mV]	Replicate 3 [mV]	Average [mV]	Std.dev [mV]
0	192.6	191.1	168.1	190.4	183.2	13.1
1	174.2	192.3	162.3	192.6	182.4	17.4
2	161.1	189.3	157.1	185.1	177.2	17.5
4	145.9	168.8	146.6	175.6	163.7	15.2
24	126.4	141.2	126.3	161.2	142.9	17.5

Repeat I: Bentonite (NM-600) tested in Gamble´s solution

Time [h]	Replicate [mV]	Replicate 1 [mV]	Replicate 2 [mV]	Replicate 3 [mV]	Average [mV]	Std.dev [mV]
0	129.6	125.2	128.9	131.5	128.5	3.17
1	135.7	115.1	128.2	127.9	123.7	7.48
2	132.8	112.6	127.3	125.8	121.9	8.09
4	127.6	107.9	124.8	121.1	117.9	8.88
24	118.3	104.1	115.0	112.0	110.4	5.63

Repeat II: Bentonite (NM-600) tested in Gamble´s solution

Time [h]	Replicate [mV]	Replicate 1 [mV]	Replicate 2 [mV]	Replicate 3 [mV]	Average [mV]	Std.dev [mV]
0	131.5	145.6	140.1	103.4	129.7	22.9
1	129.8	140.9	142.5	105.1	129.5	21.1
2	129.4	139.5	141.8	105.9	129.1	20.1
4	129.7	134.1	138.3	107.5	126.6	16.7
24	134.2	117.9	123.6	112.9	118.1	5.35

Repeat III: Bentonite (NM-600) tested in Gamble´s solution

Time [h]	Replicate [mV]	Replicate 1 [mV]	Replicate 2 [mV]	Replicate 3 [mV]	Average [mV]	Std.dev [mV]
0	132.8	125.1	133.9	141.8	133.6	8.35
1	145.1	129.0	140.1	148.2	139.1	9.64
2	142.6	130.2	140.3	148.7	139.7	9.26

4	137.4	127.4	137.4	144.0	136.3	8.36
24	124.2	110.3	121.6	122.7	118.2	6.86

Table S4. Measured redox potentials of Al_2O_3, TiO_2 (NM-104), ZnO (NM-110, NM-111, NM-113), SiO_2 (NM-200), CeO_2 (NM-212), and bentonite (NM-600) tested in phagolysosomal simulant fluid.

**Al_2O_3 tested in phagolysosomal simulant fluid**						
**Time [h]**	**Replicate [mV]**	**Replicate 1 [mV]**	**Replicate 2 [mV]**	**Replicate 3 [mV]**	**Average [mV]**	**Std.dev [mV]**
0	330.3	297.6	299.0	304.7	300.4	3.76
1	286.6	277.8	295.6	293.6	289.0	9.75
2	284.7	274.7	293.3	291.6	286.5	10.3
4	285.0	274.2	293.5	292.0	286.6	10.7
24	281.3	265.9	259.8	265.4	263.7	3.39
**TiO_2 (NM-104) tested in phagolysosomal simulant fluid**						
**Time [h]**	**Replicate [mV]**	**Replicate 1 [mV]**	**Replicate 2 [mV]**	**Replicate 3 [mV]**	**Average [mV]**	**Std.dev [mV]**
0	379.6	316.1	330.2	332.7	326.3	8.95
1	356.6	253.1	279.4	277.1	269.9	14.6
2	338.2	242.7	272.5	267.8	261.0	16.0
4	320.1	238.4	271.1	262.6	257.4	17.0
24	256.1	222.1	252.7	241.9	238.9	15.5
**ZnO (NM-110) tested in phagolysosomal simulant fluid**						
**Time [h]**	**Replicate [mV]**	**Replicate 1 [mV]**	**Replicate 2 [mV]**	**Replicate 3 [mV]**	**Average [mV]**	**Std.dev [mV]**
0	397.6	244.4	333.2	316.8	298.1	47.3
1	354.5	335.2	328.0	322.2	328.5	6.51
2	345.7	341.0	333.4	331.2	335.2	5.14
4	337.7	337.5	340.7	340.6	339.6	1.82
24	253.4	309.0	263.4	315.3	295.9	28.3
**ZnO (NM-111) tested in phagolysosomal simulant fluid**						
**Time [h]**	**Replicate [mV]**	**Replicate 1 [mV]**	**Replicate 2 [mV]**	**Replicate 3 [mV]**	**Average [mV]**	**Std.dev [mV]**
0	389.8	296.0	333.4	321.8	317.1	19.1
1	362.70	291.8	326.5	333.1	317.1	22.2
2	361.2	298.3	336.2	347.4	327.3	25.7
4	358.8	300.4	341.6	353.6	331.9	27.9
24	288.0	272.7	332.3*	308.9	193.9	30.0
**ZnO (NM-113) tested in phagolysosomal simulant fluid**						
**Time [h]**	**Replicate [mV]**	**Replicate 1 [mV]**	**Replicate 2 [mV]**	**Replicate 3 [mV]**	**Average [mV]**	**Std.dev [mV]**
0	397.4	306.1	333.7	326.5	322.1	14.3
1	368.1	313.5	333.6	345.4	330.8	16.1
2	363.2	316.0	336.3	349.7	334.0	17.0
4	356.6	319.3	339.8	350.9	336.7	16.0
24	309.1	293.8	274.0	320.1	296.0	23.1
**SiO_2 (NM-200) tested in phagolysosomal simulant fluid**						
**Time [h]**	**Replicate [mV]**	**Replicate 1 [mV]**	**Replicate 2 [mV]**	**Replicate 3 [mV]**	**Average [mV]**	**Std.dev [mV]**

0	409.6	339.0	324.9	326.1	330.0	7.82
1	377.6	317.0	311.6	317.2	315.3	3.18
2	372.0	313.2	309.1	313.9	312.1	2.59
4	361.7	305.9	304.2	308.7	306.3	2.27
24	306.5	273.1	267.9	276.4	272.5	4.29
**CeO_2 (NM-212) tested in phagolysosomal simulant fluid**						
**Time [h]**	**Replicate [mV]**	**Replicate 1 [mV]**	**Replicate 2 [mV]**	**Replicate 3 [mV]**	**Average [mV]**	**Std.dev [mV]**
0	351.2	291.7	312.0	317.6	307.1	13.6
1	329.5	262.1	298.4	293.1	284.5	19.6
2	317.1	245.7	287.8	280.0	271.2	22.4
4	300.7	231.0	276.8	266.1	258.0	24.0
24	286.7	234.1	247.2	199.1	226.8	24.9
**Bentonite (NM-600) tested in phagolysosomal simulant fluid**						
**Time [h]**	**Replicate [mV]**	**Replicate 1 [mV]**	**Replicate 2 [mV]**	**Replicate 3 [mV]**	**Average [mV]**	**Std.dev [mV]**
0	352.2	310.6	303.6	332.5	315.6	15.1
1	314.7	275.3	277.6	287.4	280.1	6.43
2	313.5	272.5	276.4	285.6	278.2	6.73
4	317.9	268.8	274.2	283.1	275.4	7.22
24	306.3	252.9	253.4	256.4	254.2	1.89

Table S5. *p*-values after Student´s t-test to evaluate potentially reactivity of the NMs in low-calcium Gamble´s solution. Significant values are highlighed in bold.

**Al_2O_3 tested in low-calcium Gamble's solution**			
**Time [h]**	**Replicate 1**	**Replicate 2**	**Replicate 3**
0	0.1421	**0.046**	0.3221
1	0.0686	**0.0142**	0.6717
2	**0.0205**	**0.0077**	0.1143
4	**0.0052**	**0.0062**	**0.0132**
24	**0.0022**	**0.0197**	**0.0114**
**TiO_2 (NM-104) tested in low-calcium Gamble's solution**			
**Time [h]**	**Replicate 1**	**Replicate 1**	**Replicate 3**
0	0.1575	**0.0082**	0.7535
1	0.1677	0.6721	0.8201
2	0.7144	0.3356	0.0831
4	0.1560	**0.0407**	**0.0167**
24	0.0154	**0.04582**	**0.0350**
**ZnO (NM-110) tested in low-calcium Gamble's solution**			
**Time [h]**	**Replicate 1**	**Replicate 1**	**Replicate 3**
0	**0.0170**	**0.0005**	**0.0027**
1	0.9184	**<0.0001**	**0.0010**
2	0.2909	**<0.0001**	**0.0035**

4	0.2736	**<0.0001**	**0.0347**
24	0.9227	**0.0109**	0.9734
**ZnO (NM-111) tested in low-calcium Gamble's solution**			
**Time [h]**	**Replicate 1**	**Replicate 1**	**Replicate 3**
0	**0.0080**	**0.0235**	**0.0128**
1	**0.0031**	**0.0240**	**0.0027**
2	**0.0077**	0.1275	**0.0038**
4	**0.0300**	0.6384	**0.0072**
24	0.4211	0.9543	0.0969
**ZnO (NM-113) tested in low-calcium Gamble's solution**			
**Time [h]**	**Replicate 1**	**Replicate 1**	**Replicate 3**
0	**0.0125**	**0.0039**	**0.0018**
1	**0.0010**	**0.0004**	**0.0002**
2	**0.0017**	**0.0008**	**0.0005**
4	**0.0065**	**0.0081**	**0.0072**
24	0.0986	0.5179	0.7137
**SiO_2 (NM-200) tested in low-calcium Gamble's solution**			
**Time [h]**	**Replicate 1**	**Replicate 1**	**Replicate 3**
0	0.1919	0.4229	0.2827
1	0.8139	0.0910	0.2645
2	0.0827	**0.0194**	0.1479
4	**0.0071**	**0.0097**	0.1364
24	**0.0016**	**0.0044**	0.2634
**CeO_2 (NM-212) tested in low-calcium Gamble's solution**			
**Time [h]**	**Replicate 1**	**Replicate 1**	**Replicate 3**
0	**0.0023**	0.8094	0.5391
1	**0.0026**	0.3134	0.1033
2	**0.0015**	**0.0230**	**0.0203**
4	**0.0016**	**0.0052**	**0.0094**
24	**0.0026**	**0.0028**	**0.0114**
**Bentonite (NM-600) tested in low-calcium Gamble's solution**			
**Time [h]**	**Replicate 1**	**Replicate 1**	**Replicate 3**
0	0.2535	0.7928	0.7790
1	**0.0006**	0.9564	0.0643
2	**0.0016**	0.9614	0.3177
4	**0.0054**	0.5425	0.6516
24	**0.0012**	**<0.0001**	**0.0161**

Table S6. p -values after Student´s t-test to evaluate potentially reactivity of the NMs in phagolysosomal simulant fluid. Significant values are highlighed in bold.

**Time**	**Al_2O_3**	**TiO_2**	**ZnO**	**ZnO**	**ZnO**	**SiO_2**	**CeO_2**	**Bentonite**

		(NM-104)	(NM-110)	(NM-111)	(NM-113)	(NM-200)	(NM-212)	(NM-600)
0	**<0.0001**	**<0.0001**	**<0.0001**	**<0.0001**	**<0.0001**	**<0.0001**	**<0.0001**	**<0.0001**
1	0.4187	**<0.0001**	**<0.0001**	**<0.0001**	**<0.0001**	**<0.0001**	**<0.0001**	**<0.0001**
2	0.5540	**<0.0001**	**<0.0001**	**0.002**	**<0.0001**	**<0.0001**	**<0.0001**	**<0.0001**
4	0.6268	**<0.0001**	**0.0062**	**0.0102**	**0.002579**	**<0.0001**	**<0.0001**	**<0.0001**
24	**<0.0001**	**0.0050**	**0.0008**	0.7460*	0.0847	**<0.0001**	**<0.0001**	**<0.0001**

*An electrode in a reactor containing ZnO (NM-111) stopped measuring after 13 hours. The point t_{24} was therefore removed.

Table S7. dE_h values in low-calcium Gamble´s solution and phagolysosomal simulant fluid.

Time	Test medium		$dE_{h\ min}$ [mV]	$dE_{h\ max}$ [mV]	Difference [mV]
Al_2O_3	Low-calcium Gamble's solution	Repeat I	-12.24	11.51	23.75
		Repeat II	-9.11	7.01	16.12
		Repeat III	-7.38	21.18	28.56
	Phagolysosomal simulant fluid		-0.58	32.11	32.69
TiO_2 (NM-104)	Low-calcium Gamble's solution	Repeat I	-9.61	19.67	29.28
		Repeat II	-0.57	32.48	33.05
		Repeat III	-9.03	13.44	22.47
	Phagolysosomal simulant fluid		-33.97	42.93	76.9
ZnO (NM-110)	Low-calcium Gamble's solution	Repeat I	-4.06	22.62	26.68
		Repeat II	-48.09	-18.09	30.00
		Repeat III	-50.24	-0.62	49.62
	Phagolysosomal simulant fluid		-4.06	22.62	26.68
ZnO (NM-111)	Low-calcium Gamble's solution	Repeat I	0.06	145.74	145.80
		Repeat II	-47.14	-6.88	40.26
		Repeat III	-35.18	5.47	40.65
	Phagolysosomal simulant fluid		-47.54	-16.83	30.71
ZnO (NM-113)	Low-calcium Gamble's solution	Repeat I	-43.66	-15.39	28.27
		Repeat II	-49.21	-6.60	42.61
		Repeat III	-48.22	-3.17	45.05
	Phagolysosomal simulant fluid		-0.91	62.94	63.85
SiO_2 (NM-200)	Low-calcium Gamble's solution	Repeat I	-11.88	26.78	38.66
		Repeat II	-10.62	29.16	39.78
		Repeat III	-9.21	4.24	13.45
	Phagolysosomal simulant fluid		-2.03	51.69	53.72
CeO_2 (NM-212)	Low-calcium Gamble's solution	Repeat I	-20.07	10.11	30.18
		Repeat II	-11.66	29.21	40.87
		Repeat III	-10.58	15.72	26.30
	Phagolysosomal simulant fluid		-21.38	4.46	25.84
Bentonite (NM-600)	Low-calcium Gamble's solution	Repeat I	-11.28	-0.01	11.29
		Repeat II	-14.20	2.38	16.58
		Repeat III	-8.31	-0.49	8.80

Phagolysosomal simulant fluid	-19.36	6.56	25.92

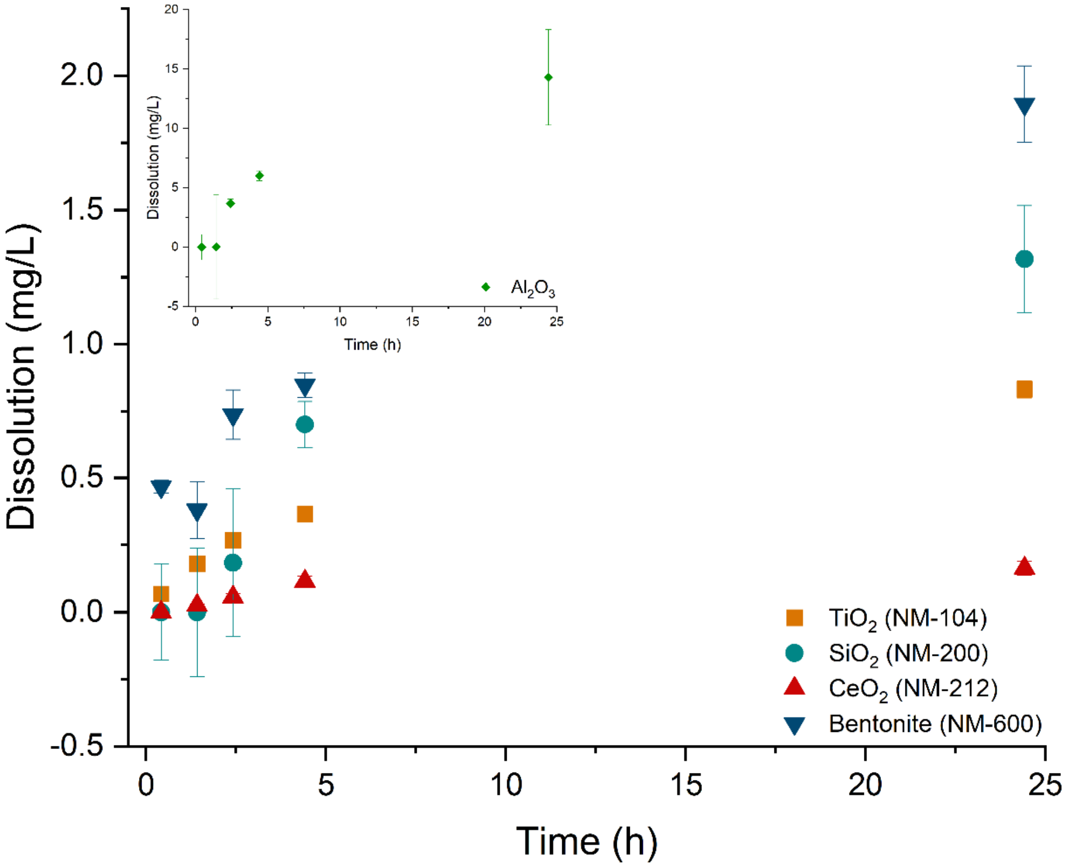

Figure S7. Depiction of dissolution in phagolysosomal simulant fluid. ZnO (NM-110, NM-111, and NM-113) is not depicted as the dissolution occurred within 25 min. Al_2O_3 (◆), TiO_2 (NM-104) (■), SiO_2 (NM-200) (●), CeO_2 (NM-212) (▲), and bentonite (NM-600) (▼).
